# The burden of young people's mental health conditions in Europe: No cause for complacency

**DOI:** 10.1016/j.lanepe.2022.100364

**Published:** 2022-03-24

**Authors:** Sharon A.S. Neufeld

**Affiliations:** Department of Psychiatry, University of Cambridge, UK

Mental health conditions in young people represent a great public health burden worldwide,^1^ yet regional-specific estimates are most informative for public health policy.^2^ In the high-income continent of Europe, mental disorders contribute a greater percentage to overall burden, despite evidence-based interventions and comparatively greater financial capacity to address these issues.^3^ Castelpietra and colleagues harnesses data from the 2019 Global Burden of Disease (GBD) study to provide estimates of the burden of mental health conditions in the 85 million young people aged 10–24 years living in 31 European countries.^4^ Epidemiological data pooled from 528 unique data sources draws attention to key areas of mental health burden in young people across Europe.

Estimating disability-adjusted life-years (DALYs) of mental disorders, substance use disorders, and self-harm helps clarify mental health priorities in European young people more than prevalence rates alone. DALYs measure the gap between current health and standard life expectancy spent in full health.^3^ DALYs are based on the sum of years lived with disability (YLD), which take into account both prevalence and severity of the condition, and years of life lost (YLL) due to premature mortality.^4^ DALYs due to mental disorders and substance use disorders largely result from disability burden (YLDs), whereas self-harm DALYs are chiefly due to premature deaths (YLLs). Castelpietra and colleagues bring together these three leading causes of disability in young people which are classed in different categories within the GBD framework.^1^ Gender differences in DALYs were stark: young women's DALYs were predominated by depression and anxiety, but in young men, these two conditions had equivalent DALYs to drug use disorders and self-harm.^4^ However, when only prevalence was considered, the priorities in males changed, as anxiety and ADHD were the most prevalent. Across the age groups studied, further patterns emerged. For example, in 20-24-year old males, DALYs were significantly greater for self-harm and drug use disorders, yet prevalence in this group was led by anxiety, alcohol use and drug use disorders (Figure 1). By combining prevalence, severity, and YLLs in DALYs, Castelpietra and colleagues’ data highlights differential mental health burdens by age and gender. This can help focus young people's mental illness prevention programs on conditions which have the greatest impact on young people’s lives, and appropriately tailor interventions during this sensitive period of development.

**Figure 1:**
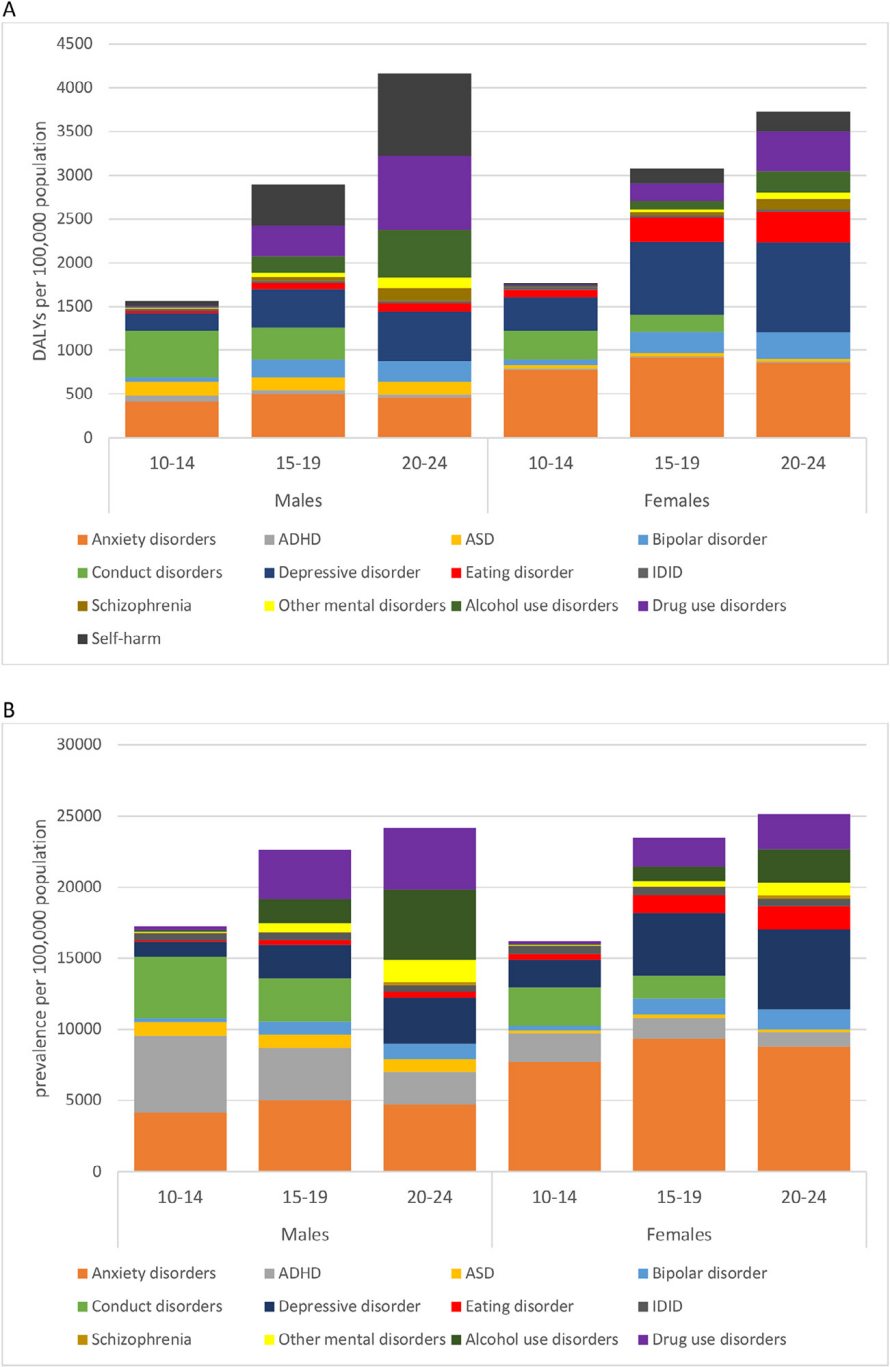
Disability-adjusted life-years (DALYs; panel A) and prevalence (panel B) of mental disorders, substance use disorders, and self-harm from the Global Burden of Disease Study 2019; data by age and gender, from Castelpietra et al, 2022. ADHD=Attention deficit/hyperactivity disorder; ASD=Autism spectrum disorders; IDID=Idiopathic developmental intellectual disability. Self-harm not included in panel B as only incidence estimates were provided, since the vast majority of burden due to self-harm was represented by years of life lost due to fatal self-harm.

Child and adolescent mental health services (CAMHS) capacity across European countries^2^ does not appear to map onto subsequent rates of mental health conditions reported by Castelpietra and colleagues. For instance, per 100 000 young people in European countries, despite having one of the highest levels of CAMHS in 2013-14, the UK had the highest prevalence of severe mental disorders and drug use disorders in 2019.^4^ While Eastern European countries appeared to have the lowest prevalence of mental health conditions in 2019, this was not reflected in high levels of CAMHS capacity,^2^ and prevalence may be underestimated due to less available data from Eastern European countries.^4^ CAMHS effectiveness is driven by a complex interplay between the availability of services and specialists, financing, collaboration with other services, and reporting standards.^2^ This complexity points to the need for a rigorous study of the relationship between these various facets of CAMHS and the burden of mental health conditions in young people to highlight ways in which CAMHS can be improved across various European countries.

Does knowledge of the prior 30-year trends in mental health conditions help us as we face the mental health consequences of the COVID-19 pandemic? Castelpietra and colleagues note that of all conditions studied, the largest increase from 1990-2019 occurred in eating disorders – a 15% increase in YLDs across the full sample, with YLDs increasing in both genders across all age groups studied, and YLLs significantly increasing for females aged 15–24. This is troubling, especially considering that GBD studies underestimate the burden of eating disorders, being based on outdated diagnostic classification systems which exclude relevant disorders such as binge-eating.^5^ Along with the significant increase in eating disorders observed in young females in the COVID-19 pandemic,^6^ this sets the stage for eating disorders becoming a major mental health burden in European young people. From 1990-2019, YLDs for drug use disorders increased at a comparable rate to eating disorders (seen in both genders in 15–24 year-olds) whilst YLLs declined by a comparable amount (driven by males aged 20-24).^4^ This shift from YLLs to YLDs could be related to interventions to reduce drug-related deaths, such as supervised drug consumption and opioid substitution treatment.^7^ Increased emphasis on effective interventions is key to minimising the burden from eating and substance use disorders.

Finally, over the 30-year period Castelpietra and colleagues observed a 28% decline in YLL due to self-harm – a decline observable in both genders and all ages studied, peaking at over 50% for boys aged 10-14. Most of the burden was due to YLL from fatal self-harm^8^ which in 2019 still represented a substantial burden, contributing the greatest number of premature deaths by far. This overall decline was comparable to global reports of self-harm in 10-24-year olds^1^ and consistent with global trends in suicide.^9^ The decline seen in European young people could be partially attributable to the concomitant decline in prevalence of alcohol use disorders,^4^ a predictor of self-harm.^8^ Improved access to mental health services or suicide prevention strategies may have also contributed to this decline.^2^^,^^9^ However, looking forward, the COVID-19 pandemic has resulted in a global increase in anxiety and major depressive disorder in young people,^10^ both risk factors for self-harm.^8^ Eating disorders and substance use disorders are also risk factors^8^ and thus their increase prior to the pandemic^4^ may also contribute to increased self-harm. Furthermore, during the pandemic, eating disorders have been more strongly associated with suicidality than previously.^6^ Therefore, self-harm should remain a prominent focus of mental illness prevention strategies across Europe.

Continued work is required to improve prevention efforts, practice, and service delivery to relieve the burden associated with mental health conditions in young people in Europe. Despite many competing needs arising from the COVID-19 pandemic, we must urge policymakers to keep mental health a priority. Particularly after the past two years, our young people deserve far more than complacency.

## Author contributions

SN conceived the arguments expressed, drafted, and critically revised the manuscript.

## Declaration of interests

Dr. Neufeld has nothing to disclose.
